# A cell culture platform for quantifying metabolic substrate oxidation in bicarbonate-buffered medium

**DOI:** 10.1016/j.jbc.2021.101547

**Published:** 2021-12-29

**Authors:** James R. Krycer, Mary Lor, Rebecca L. Fitzsimmons, James E. Hudson

**Affiliations:** 1 QIMR Berghofer Medical Research Institute, Brisbane, Queensland, Australia; 2 Faculty of Health, School of Biomedical Sciences, Queensland University of Technology, Brisbane, Queensland, Australia; 3 Faculty of Medicine, School of Biomedical Sciences, The University of Queensland, Brisbane, Queensland, Australia

**Keywords:** gas trap, cell metabolism, oxidation, carbon dioxide, bicarbonate, glucose, adipocyte, BSA, bovine serum albumin, BSF-DMEM, bicarbonate-buffered, serum-free DMEM, DMEM, Dulbecco’s modified Eagle medium, HSF-DMEM, HEPES-buffered, serum-free DMEM, PETG, polyethylene terephthalate glycol

## Abstract

Complex diseases such as cancer and diabetes are underpinned by changes in metabolism, specifically by which and how nutrients are catabolized. Substrate utilization can be directly examined by measuring a metabolic endpoint rather than an intermediate (such as a metabolite in the tricarboxylic acid cycle). For instance, oxidation of specific substrates can be measured *in vitro* by incubation of live cultures with substrates containing radiolabeled carbon and measuring radiolabeled carbon dioxide. To increase throughput, we previously developed a miniaturized platform to measure substrate oxidation of both adherent and suspension cells using multiwell plates rather than flasks. This enabled multiple conditions to be examined simultaneously, ideal for drug screens and mechanistic studies. However, like many metabolic assays, this was not compatible with bicarbonate-buffered media, which is susceptible to alkalinization upon exposure to gas containing little carbon dioxide such as air. While other buffers such as HEPES can overcome this problem, bicarbonate has additional biological roles as a metabolic substrate and in modulating hormone signaling. Here, we create a bicarbonate-buffered well-plate platform to measure substrate oxidation. This was achieved by introducing a sealed environment within each well that was equilibrated with carbon dioxide, enabling bicarbonate buffering. As proof of principle, we assessed metabolic flux in cultured adipocytes, demonstrating that bicarbonate-buffered medium increased lipogenesis, glucose oxidation, and sensitivity to insulin in comparison to HEPES-buffered medium. This convenient and high-throughput method facilitates the study and screening of metabolic activity under more physiological conditions to aid biomedical research.

Metabolic dysregulation is now emerging as a common feature of numerous complex diseases such as cancer (1), inflammatory disorders (*e.g.*, (2)), and cardiovascular disease (3). A key aspect of metabolism research is understanding which specific substrates are oxidized to CO_2_. For instance, the heart is capable of oxidizing both glucose and fatty acids, but a preference for fatty acids develops during maturation (4, 5). Indeed, glucose and fatty acid utilization are reciprocally regulated across multiple tissues to maintain energy homeostasis, with perturbations in this balance observed in metabolic diseases such as diabetes (6). Thus, it is critical to understand how the oxidation of different substrates varies between tissues and with disease.

## Measuring substrate oxidation *in vitro*

There are several methods available for assessing substrate oxidation. For instance, the consumption of oxygen can be measured with respirometry. Intact cells or isolated mitochondria are incubated with specific substrates, followed by pharmacological inhibition of key enzymes. This enables the cell’s capacity to oxidize a particular substrate to be inferred, but cannot dissect substrate preference in complex environments. For instance, insulin markedly increases glucose uptake and oxidation in adipocytes (7, 8), but insulin-stimulated respiration is unaffected by the removal of glucose (9). This implies that in the absence of glucose, adipocyte respiration compensates by oxidizing other substrates such as amino acids. Thus, respirometry may not resolve the oxidation of specific substrates under physiological conditions, where multiple substrates are present. This limitation can be overcome with metabolic labeling. A substrate of interest can be ^13^C-labeled, and the incorporation of substrate carbon into metabolites in the tricarboxylic acid cycle can be assessed by mass spectrometry or nuclear magnetic resonance. However, metabolic flux rates are more difficult to quantify under acute, non-steady-state conditions (10) and are an indirect measure of oxidation since most tricarboxylic acid cycle metabolites participate in other metabolic pathways.

A direct means of quantifying oxidation is by measuring the incorporation of isotopically labeled substrates into CO_2_. Here, substrates are labeled with ^14^C instead of ^13^C due to the comparative ease and sensitivity of liquid scintillation counting. The general methodology involves (1) incubating with ^14^C-labeled substrate, (2) after the experiment, adding a strong acid to quench metabolism and liberate CO_2_, (3) trapping the CO_2_ generated with an alkali solution, and (4) measurement of ^14^C signal in the trapped CO_2_ by liquid scintillation counting. Historically, this involved incubation of cells or tissues in sealed flasks or vials, with the alkali solution held in a separate vessel within the flask/vial such as in a “hanging well” attached to the stopper (*e.g.*, (8, 11, 12)). Alternatively, an aliquot of conditioned medium is transferred to a second vial, where the medium is acidified and CO_2_ is trapped (*e.g.*, (13)).

## A microplate-based, bicarbonate-compatible method to measure substrate oxidation

To enable cell culture perturbation and drug testing, we previously developed a platform for measuring substrate oxidation in multiwell plates (14). In 12-well plates, the alkali solution was held in perforated plastic lids. The rubber stopper used traditionally to seal flasks was substituted with a gas-impermeable plate seal. This only required general lab consumables and a drill, making this platform cost-effective and accessible. This enabled the assessment or screening of substrate oxidation in multiwell plates for both adherent and suspension cells (14) and *Drosophila* (15).

Using this platform, bicarbonate needed to be replaced with HEPES due to lack of gas-phase CO_2_ for buffering. This is common practice, with bicarbonate replaced with HEPES in respirometry (*e.g.*, (16)) and metabolic assays (*e.g.*, (17, 18, 19, 20, 21, 22)). Since HEPES is a Good’s buffer (23), HEPES-buffered media still facilitate robust results from *in vitro* experiments without needing CO_2_ buffering. However, bicarbonate can influence metabolism beyond its role as an extracellular pH buffer (24). Bicarbonate acts as a metabolic substrate for carboxylation reactions, such as those catalyzed by acetyl-CoA carboxylase in lipogenesis or pyruvate carboxylase in gluconeogenesis. By interconverting with CO_2_, bicarbonate also mediates intracellular pH fluctuations, which have been demonstrated to influence glycolysis (25, 26). Indeed, pH can influence the regulation of enzymes, such as lactate dehydrogenase (27), and proton-dependent transporters, such as monocarboxylate transporters such as MCT1 that couple lactate and proton efflux (28). Bicarbonate can also impact cell signal transduction, highlighted by the sensitivity of soluble adenylyl cyclase to bicarbonate (29). Thus, compared with other pH buffers, bicarbonate uniquely acts in multiple ways to support metabolism.

Here, we adapted our plate-based protocol to be compatible with a bicarbonate-buffered environment. We present an accessible and convenient platform using general lab equipment and consumables as a means of achieving this outcome. Applying this new protocol to cultured adipocytes, we demonstrate that bicarbonate increases not only glucose oxidation and lipogenesis, but also the sensitivity to insulin. This platform therefore enables the study of metabolism in well-plates under more physiological conditions to facilitate the study and screening of metabolism for biomedical research.

## Results

### Development of the gas manifold

**Figure 1:**
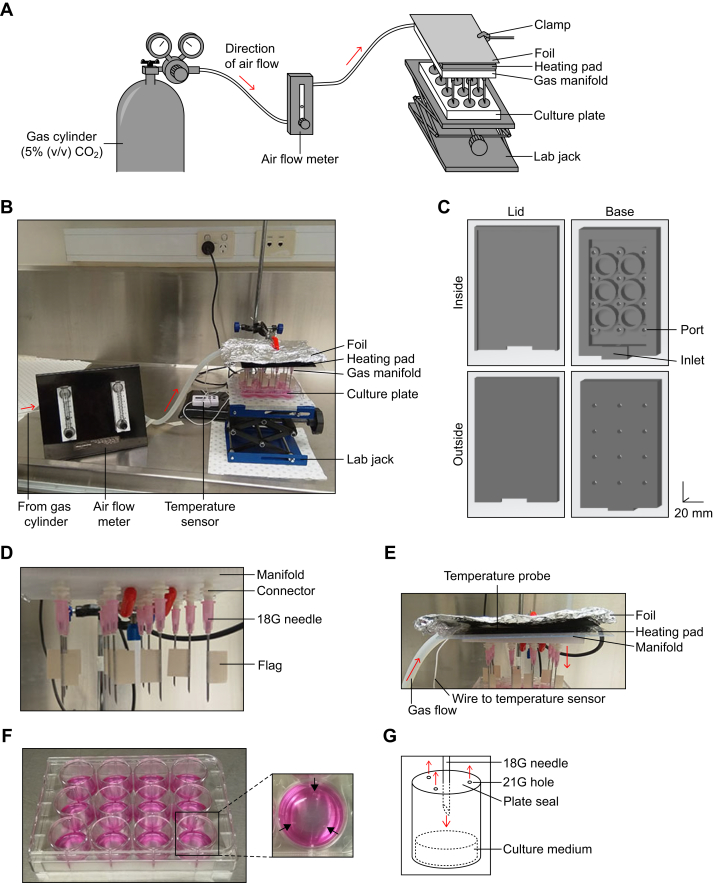
**Overview of the heated gas manifold.***A*, schematic of the heated gas manifold. The clamp is attached to a retort stand, which is not shown. An electronic temperature sensor embedded between the foil and heat pad is also not shown. Further details for each component are available in the main text and Supporting information. *B*, photo depicting the heated gas manifold. Direction of gas flow indicated by *red arrows*. Gas cylinder is not shown. Further details for each component are available in the main text and Supporting information. *C*, schematic of the manifold, printed separately as a lid and base. Scale shown in the *bottom right*, for the x-, y-, and z-directions. *D*, close-up of the underside of the manifold, depicting the male–male luer slip connectors and needles. *E*, close-up of the side of the manifold, depicting the heating pad above the manifold. The temperature probe is in between the heating pad and foil. *F*, photo depicting a sealed culture plate, with each well containing 500 μl of BSF-DMEM supplemented with 15 mg/l phenol red and exposed to air for at least 30 min. Close-up depicts three equidistant holes produced by puncturing the seal with a beveled 21G needle. *G*, schematic depicting the principle behind the gas equilibration, detailed in the main text. BSF-DMEM, bicarbonate-buffered, serum-free Dulbecco’s modified Eagle medium.

To make our microplate-based substrate oxidation assay compatible with bicarbonate, we needed to maintain a gas phase of 5% (v/v) CO_2_ within each well. To achieve this, we constructed a heated gas manifold (Fig. 1, *A* and *B*). The manifold was printed with polyethylene terephthalate glycol (PETG) and contained 12 ports (Fig. 1*C*), one for each well. Each port was designed to fit a male–male luer slip connector and 18G needle (Fig. 1*D*). A masking-tape “flag” was glued to each needle to control the depth that the needle entered the well. The manifold was warmed using a heating pad covered in aluminum foil (Fig. 1*E*), producing a temperature of approximately 40 °C directly above the manifold when gas flowed through the manifold. To equilibrate the culture plate, the plate was sealed and three equidistant holes were punctured with a 21G needle (Fig. 1*F*). This permitted gaseous exchange when the gas phase was equilibrated by the manifold (Fig. 1*G*). A second layer of plate seal was then immediately applied to the culture plate to prevent the loss of gas-phase CO_2_.

### Optimization of the gas manifold

**Figure 2:**
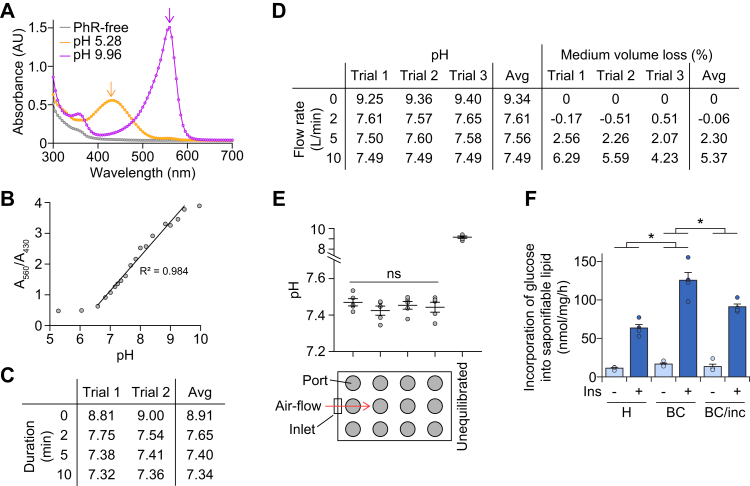
**Optimization of the gas manifold.***A* and *B*, BSF-DMEM was supplemented with 15 mg/l phenol red (*PhR*) and titrated to the indicated pH’s using either 1 M NaOH or HCl. At each pH, an aliquot was stored in a sealed microfuge tube (with minimal air volume). In *A*, for each sample at the indicated pH (or BSF-DMEM without PhR), 100 μl was pipetted into duplicate wells of a 96-well plate and absorbance at 300 to 700 nm was immediately measured by spectrophotometry. Absorbance maxima indicated by *arrows*. In *B*, for one sample (pH) at a time, 500 μl was pipetted into duplicate wells of a 12-well microplate, and absorbance was immediately measured at 430 and 560 nm. The ratio of absorbance is plotted against pH. *C*, using BSF-DMEM supplemented with 15 mg/l PhR, 500 μl was pipetted into each well of four 12-well microplates. Media were alkalinized by exposure to air at room temperature for at least 30 min, before plates were sealed and equilibrated with 5% (v/v) CO_2_ for the indicated durations. Following equilibration, plates were resealed and absorbances at 430 and 560 nm were measured after 15 min. The line of best fit in *B* was used to convert absorbance ratio to pH for each well, with the average pH across the plate presented for each trial. *D*, the experiment was performed as in *C*, except with the indicated flow rates for 5 min. Furthermore, plates were weighed before and after addition of media, and after absorbance measurement, to calculate media volume loss. *E*, using the data in *C* and *D*, the medium pH after gas equilibration at 10 l/min for 5 min is presented as the average across each column of the plate, with the *leftmost column* reflecting wells closest to the gas inlet. The medium pH of unequilibriated medium (averaged across the whole plate) is included as a control. Data presented as mean ± SEM, with each data point reflecting an individual experiment. ns, *p* > 0.05 by one-way ANOVA comparing the effect of column number (distance from inlet) on medium pH. Experiments in *A*–*E* were performed at room temperature to minimize the impact of temperature fluctuations. *F*, 3T3-L1 adipocytes were serum-starved and treated with or without 100 nM insulin (*Ins*), in either: *H*: HEPES-buffered medium, with plates sealed prior to incubation at 37 °C, *BC*: bicarbonate-buffered medium, with plates sealed, gas-equilibrated, and resealed prior to incubation at 37 °C, or *BC/inc*: bicarbonate-buffered medium, with plates left unsealed in a humidified incubator at 37 °C and 5% (v/v) CO_2_. After 60 min, cells were quenched and saponifiable lipids were extracted as described in the Experimental procedures. The incorporation of ^14^C-glucose into the saponifiable lipid fraction was assessed by liquid scintillation counting. Data presented as mean + SEM, from four separate experiments. ∗*p* < 0.05 by two-way ANOVA comparing the effect of buffer system (*BC versus H*, *BC versus BC/inc*) on glucose incorporation into saponifiable lipid. BSF-DMEM, bicarbonate-buffered, serum-free Dulbecco’s modified Eagle medium.

We optimized the gas flow parameters using naïve culture medium, bicarbonate-buffered Dulbecco’s modified Eagle medium (DMEM). The medium was alkalinized by prolonged exposure to air (to pH ∼ 9) and then equilibrated with 5% (v/v) CO_2_ to restore the medium pH to 7.4 ± 0.1. The medium pH was measured using phenol red, a pH indicator which produced absorbance maxima at 560 nm (alkali) and 430 nm (acidic) (Fig. 2*A*). The ratio of these absorbances exhibited a linear correlation with pH between pH 6.5 and 9.5 (Fig. 2*B*), allowing us to determine medium pH by spectrophotometry. With a gas flow of 10 l/min, we found that 5 min was sufficient for equilibration (Fig. 2*C*). Lower flow rates failed to reduce the pH to 7.4 within this timeframe (Fig. 2*D*). We reasoned that equilibration at 10 l/min for 5 min provided the best balance between time and flow because the volume loss was negligible (Fig. 2*D*). We checked that the gas equilibration was consistent between wells. It is possible that the ports further from the inlet experienced a lower gas flow, leading to their respective wells receiving less 5% (v/v) CO_2_ and not being properly equilibrated. However, distance from the inlet had no impact on medium pH (Fig. 2*E*). Given the variation between 3D printers, printing materials and culture medium, we recommend optimizing these parameters for each individual manifold.

We applied our optimized gas-flow conditions to cultured adipocytes and measured lipogenesis, which utilizes bicarbonate as a substrate for carboxylation. As a control, we used HEPES-buffered DMEM without equilibration with 5% (v/v) CO_2_. Upon insulin stimulation, more glucose was incorporated into saponifiable lipid (*e.g.*, fatty acids) when adipocytes were cultured in bicarbonate-buffered medium compared with HEPES-buffered medium (Fig. 2*F*, *H versus BC*). Lipogenesis was also slightly higher with our sealed system compared with a CO_2_ incubator (Fig. 2*F*, *BC versus BC/inc*). Thus, the gas manifold enabled us to achieve buffered bicarbonate medium in a (sealed) microplate for metabolic experiments.

### Adapting gas trapping for bicarbonate buffers

**Figure 3:**
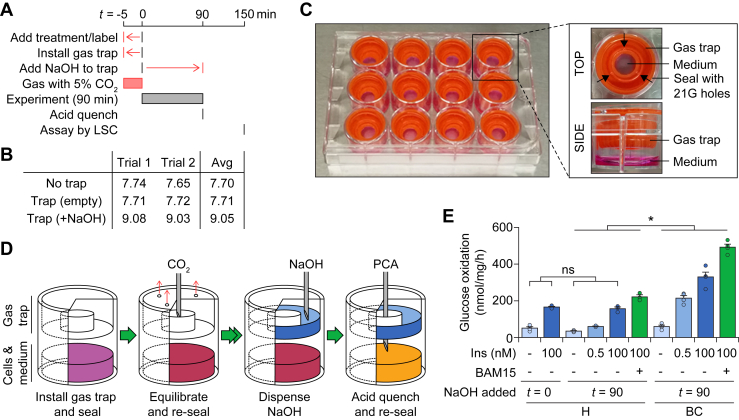
**Application of the gas manifold to the gas-trapping assay.***A*, schematic depicting how the existing gas-trapping protocol (14) can be modified to accommodate CO_2_ equilibration. Modifications shown in *red*. Details in the main text. *B*, the experiment was performed using culture medium as described in Figure 2*C*. For wells with a gas trap, an empty gas trap was installed, and the entire plate was sealed before gas equilibration for 5 min at 10 l/min. The plate was resealed and NaOH was loaded into the gas trap using a 27G needle, with identical treatment (without NaOH in the syringe) for the control wells. The plate was again resealed before incubation at room temperature for 60 min. Absorbance ratios were measured and converted to pH using the line of best fit in Figure 2*B*, with the average pH across replicates (on the same plate) presented for each trial. The deviation in pH from 7.4 in the control wells was due to the extra time taken, to remove the gas traps, between removing the plate seal and measuring absorbances. *C*, photo depicting a sealed culture plate with gas traps installed. Each well contains 500 μl of BSF-DMEM supplemented with 15 mg/l phenol red and exposed to air for at least 30 min. Top-view close-up shows three equidistant holes punctured by a 21G needle, whereas side-view close-up shows the relative positions of the gas trap and medium. For the side view, the neighboring wells were emptied for clarity. *D*, schematic depicting the bicarbonate-compatible CO_2_-trapping protocol. Details in the main text. *E*, 3T3-L1 adipocytes were treated with the indicated insulin (*Ins*) treatments with or without 10 μM BAM15, in either HEPES- (*H*) or bicarbonate (*BC*)-buffered DMEM. The experiment was performed as described in Figure 2*F*, except gas traps were installed prior to the start of the experiment (*H*) or gas equilibration (*BC*). NaOH was added to the gas trap either before the experiment (*t* = 0) or after the experiment before acid quenching (*t* = 90). This protocol is depicted in *A* and *D*, and described in the Experimental procedures and Supporting information. After 90 min of treatment, cells were quenched by PCA and CO_2_ incorporation into the NaOH solution was measured by liquid scintillation counting. Data presented as mean + SEM from three (*H*) or four (*BC*) separate experiments. ns, *p* > 0.05 by two-way ANOVA comparing the effect of the timing of NaOH addition on measured glucose oxidation (utilising four conditions only: all H buffer, either *t* = 0 or 90, either no insulin or 100 nM insulin). ∗, *p* < 0.05 by two-way ANOVA comparing the effect of buffer system (*BC versus H*) on glucose oxidation (utilizing eight conditions: all *t* = 90, either H or BC buffer, every drug treatment). BSF-DMEM, bicarbonate-buffered, serum-free Dulbecco’s modified Eagle medium; LSC, liquid scintillation counting; PCA, perchloric acid.

Next, we adapted our CO_2_-trapping methodology (14) to accommodate a bicarbonate-buffered medium (Fig. 3*A*). Previously, at the start of the experiment, a gas trap was installed and filled with NaOH before the culture plate was sealed (14). Adopting this approach promoted alkalinization of bicarbonate-buffered medium (Fig. 3*B*). We circumvented this by installing an empty gas trap before equilibration with 5% (v/v) CO_2_ (Fig. 3*C*), only dispensing NaOH into the gas trap at the end of the treatment, just prior to quenching with acid (Fig. 3, *A* and *D*). A detailed version of this protocol is available in Supporting information (Appendix 1).

### Bicarbonate influences adipocyte metabolism

We applied our protocol to measure glucose oxidation in cultured adipocytes (Fig. 3*E*), treated with insulin to stimulate glucose oxidation. First, we compared the addition of NaOH at the start (*t* = 0) or end (*t* = 90) of the treatment period, in HEPES-buffered medium (without CO_2_ equilibration). We found no difference in glucose oxidation in either control- or insulin-treated cells (Fig. 3*E*, *H: t* = 0 *versus* 90), confirming that CO_2_ trapping after quenching is sufficient to capture all the products of oxidation. In parallel, we treated adipocytes in bicarbonate-buffered media (with CO_2_ equilibration), finding that bicarbonate increased insulin-stimulated glucose oxidation (Fig. 3*E*, *BC versus H*). The oxidation rate upon stimulation with 0.5 nM insulin was 57.3 ± 4.0% that of 100 nM insulin in bicarbonate-buffered medium and only 21.0 ± 4.3% in HEPES-buffered medium (Fig. 3*E*). This demonstrates that bicarbonate increased the sensitivity of adipocytes to insulin. Additionally, when treated with the mitochondrial uncoupler BAM15 to induce maximal substrate oxidation (7), glucose oxidation was higher in bicarbonate-buffered medium in comparison to HEPES (Fig. 3*E*), revealing that bicarbonate also enhanced the capacity for glucose oxidation. Overall, this demonstrated that adipocyte metabolism is strongly influenced by the presence of bicarbonate.

## Discussion

In this study, we developed a gas-trapping method that enables the oxidation of metabolic substrates to be measured in cell culture plates, in the presence of bicarbonate-buffered medium. We achieved this using a heated gas manifold (Figs. 1 and 2) and substantial method optimization (Figs. 2 and 3). Applying this protocol to adipocytes, we found that bicarbonate increased glucose oxidation (Fig. 3*E*), glucose incorporation into saponifiable lipid (Fig. 2*F*), and sensitivity to insulin (Fig. 3*E*). Overall, this highlights the importance of bicarbonate as a medium additive in metabolic experiments.

Making a microplate-based protocol compatible with a gas phase of 5% (v/v) CO_2_ involved overcoming several challenges. First, a sealed environment needed to be maintained. We found that a gas-impermeable plate seal conveniently enabled all wells to be sealed simultaneously. Also, the equilibration of media was more consistent if the plate was sealed before rather than after equilibration (not shown). Consequently, our protocol (Fig. 3, *A* and *D*) involved repeatedly puncturing the seal with needles and subsequent resealing. This resulted in minimal loss of CO_2_ since the medium pH reached ∼7.4 following equilibration (Fig. 2, *C* and *D*). Furthermore, adipocytes in the sealed plate incorporated more glucose into saponifiable lipid than those in a CO_2_ incubator (Fig. 2*F*). Since this metabolic reaction was bicarbonate-sensitive (Fig. 2*F*, HEPES *versus* bicarbonate), this was likely due to the slower equilibration of medium bicarbonate in the incubator and fluctuations in gas-phase CO_2_ levels caused by opening the incubator during an experiment.

Second, the gas-trapping solution (NaOH) could not be in the presence of 5% (v/v) CO_2_. Theoretically, the quantity of NaOH we used was sufficient to absorb the CO_2_ from the bicarbonate-buffered medium, gas phase, and substrate oxidation (Supporting information, Appendix 2). This is necessary for gas trapping, but also implies that NaOH is capable of alkalinizing the medium if present during an experiment (Fig. 3*B*). To avoid this, we dispensed the NaOH at the conclusion of the experiment (Fig. 3, *A* and *D*). The 60 min trapping period was still sufficient (Fig. 3*E*) and substantially longer than previous protocols (*e.g.*, 15 min (8)).

Third, gas equilibration needed to occur without substantial cooling or evaporation of the culture medium. The former was circumvented by warming the gas with a heating pad and prewarming the culture medium, and the latter was mitigated by optimizing the gas flow parameters (Fig. 2). Although some evaporation was measured, this was minimal and can be adjusted for by preparing a diluted medium—for instance, if a 5% evaporation rate is consistently observed, the medium could be prepared in 1.05 volume. Alternatively, the CO_2_ may be humidified, but this requires maintaining the gas tubing in a warm environment to prevent condensation. Overall, we present a protocol that is convenient, cost-effective, and can be easily tailored to individual experiments.

As proof of principle, we examined the influence of bicarbonate on adipocyte metabolism. Bicarbonate increased lipogenesis (Fig. 2*F*) and glucose oxidation (Fig. 3*E*), likely because bicarbonate facilitates intracellular pH fluctuations and participates as a metabolic substrate. For instance, bicarbonate is required by the acetyl-CoA carboxylase enzymes; pharmacological (30) or genetic inhibition (31) of these enzymes impairs lipogenesis. Our data concur with previous observations that bicarbonate impacts mitochondrial respiration (32, 33). Furthermore, bicarbonate increased sensitivity to insulin, which has been observed previously in isolated primary adipocytes (34). In fact, the decrease in insulin responsiveness upon replacing bicarbonate is a comparable effect size to the impact of insulin resistance on glucose consumption (7). By affecting insulin action, this highlights that bicarbonate impacts signal transduction pathways beyond cAMP-dependent kinases (29). Together, our data support the notion that bicarbonate serves numerous roles in cellular physiology. This may explain why bicarbonate is advantageous over HEPES in functional experiments of tissues *ex vivo*, such as in cardiac tissue (35).

Overall, our gas-trapping protocol permits the study of substrate oxidation under modern cell culture conditions. As a microplate assay, it is suitable for adherent or suspension cells (14) and facilitates high-volume experiments. Importantly, it is compatible with medium buffered with bicarbonate, the endogenous buffering system that influences metabolism and hormonal signaling in multicellular organisms. Overall, this presents a convenient and high-throughput method for the study of metabolism under more physiological conditions.

## Experimental procedures

A more detailed version of the protocol for constructing and using the gas manifold for substrate oxidation assays is available in the Supporting information (Appendix 1). No human and animal experiment investigations were performed in this study.

### Construction of gas manifold

The gas manifold was designed using TinkerCAD (Autodesk; https://www.tinkercad.com/things/fGvoG1pJUVb) consisting of a lid and base (Fig. 1*C*). Both parts were printed with PETG (Dremel Digilab, catalog no. PETG-TRA-01) using the 3D45 3D printer (Dremel Digilab) with a nozzle temperature of 250 °C, build platform temperature of 70 °C, and print speed of 60 mm/s. The lid and base were adhered and sealed using silicon sealant (RS Components, catalog no. 555-588). Each port was fitted with a nylon male–male luer-slip connector (Cole-Parmer, catalog no. 45505-72; distributed by John Morris Scientific, catalog no. 1076298), with the manifold end of the connector coated with super glue (Tarzan’s Grip; Bunnings Warehouse, catalog no. 1230185) (Fig. 1*D*). The other end of the connector was fitted with a beveled 18G needle (1.2 × 38 mm; Terumo TMP, catalog no. AN-1838R1). Each needle had a small piece of masking tape attached with super glue, 1 cm from the tip, to prevent the needle from piercing too deeply into the well.

Using silicon tubing, the inlet of the manifold was connected *via* an air-flow meter (Dwyer Instruments, catalog no. 172989-00) to a G2 gas cylinder containing 5% (v/v) CO_2_ and 21% (v/v) O_2_ in N_2_ (BOC, catalog no. LSA400011G2) (Fig. 1, *A* and *B*). The manifold was heated using a 5 W heating pad (Reptile One Heat Mat 240 V, 14 × 15 cm; PetBarn, catalog no. 46527) covered with aluminum foil (Fig. 1*E*), all clamped to a retort stand. An electronic temperature gauge (Jaycar Electronics, catalog no. QM7209) was inserted between the heating pad and foil for temperature monitoring. The heating pad was switched on at least 30 min prior to experiments that required heated gas (*e.g.*, cell culture experiments).

### Gas-phase equilibration of cell culture plates

Following dispensing of media into the cell culture microplate, the plate was sealed with TopSeal-A PLUS (PerkinElmer, catalog no. 6050185), trimmed to size. Three equidistant holes were made approximately 0.5 mm from the well edge using a beveled 21G needle (0.8 × 38 mm; Terumo TMP, catalog no. AN-2138R1) (Fig. 1*F*). The plate was then lifted up to the gas manifold such that the manifold needles punctured the seal in the middle of each well. The plate was held in place using a laboratory jack (Eisco 150 × 135 mm; WestLab, catalog no. 071210-0001). The gas phase was equilibrated at 10 l/min for 5 min, unless otherwise specified in the figure legends. After equilibration, the plate was removed from the manifold and another layer of plate seal was immediately applied. The plate was incubated as described in the figure legends.

### Reagents for cell culture experiments

The following reagents were used in this study: dexamethasone (Sigma-Aldrich, catalog no. D4902), biotin (Sigma-Aldrich, catalog no. B4639), insulin (human recombinant; Thermo Fisher, catalog no. 12585014), 3-isobutyl-1-methylxanthine (Sigma-Aldrich, catalog no. I7018), fatty-acid free bovine serum albumin (fatty-acid free BSA; Sigma-Aldrich, catalog no. A7030), [U-^14^C]-glucose (PerkinElmer, catalog no. NEC042X001MC), BAM15 (Sigma-Aldrich, catalog no. SML1760).

### Cell culture

3T3-L1 fibroblasts were a gift from Prof. George Muscat (Institute of Molecular Bioscience, University of Queensland). 3T3-L1 fibroblasts were passaged at 50 to 60% confluency in Medium A, consisting of DMEM (Sigma-Aldrich, catalog no. D1145) supplemented with 10% (v/v) fetal bovine serum (Thermo Fisher Scientific, catalog no. 10099141), 2 mM GlutaMAX (Thermo Fisher Scientific, catalog no. 35050061), and 100 U/ml penicillin and streptomycin (Thermo Fisher Scientific, catalog no. 15140122). After reaching 100% confluency, 3T3-L1 fibroblasts were differentiated into adipocytes first by incubation in Medium A supplemented with 250 nM dexamethasone, 400 nM biotin, 2 μg/ml insulin (∼350 nM), and 0.5 mM 3-isobutyl-1-methylxanthine. After 3 days, the medium was replaced with Medium A supplemented with 2 μg/ml insulin. After 3 days, adipocytes were maintained in Medium A. Cell cultures were maintained at 37 °C in a humidified atmosphere with 5% (v/v) CO_2_. Cells were routinely tested for *Mycoplasma* contamination.

Adipocytes were used for experiments between days 9 and 12 after the initiation of differentiation, with at least 90% of the cells differentiated into adipocytes. Experiments were conducted in 12-well plates (Corning Costar, distributed by Sigma-Aldrich, catalog no. CLS3513). Prior to the experiment, adipocytes were washed thrice with PBS and serum-starved for at least 2 h in Medium B, which consisted of DMEM (Sigma-Aldrich, catalog no. D1145) supplemented with 0.2% (w/v) fatty-acid free BSA and 2 mM GlutaMAX. Following serum starvation, adipocytes were either treated in bicarbonate- or HEPES-buffered media, as outlined below.

For incubation in bicarbonate-buffered medium, adipocytes were washed once with PBS and once with bicarbonate-buffered, serum-free DMEM (BSF-DMEM), which consisted of DMEM (Sigma-Aldrich, catalog no. D5030) supplemented with 44 mM NaHCO_3_ and adjusted to pH 7.4. Cells were then incubated in 500 μl of BSF-DMEM supplemented with 0.2% (w/v) fatty-acid free BSA, 5 mM glucose, 1 μCi/ml [U-^14^C]-glucose, 2 mM glutamine, and 15 mg/l phenol red. Alternatively, for incubation in HEPES-buffered medium, adipocytes were treated similarly except BSF-DMEM was replaced with HEPES-buffered, serum-free DMEM (HSF-DMEM), which consisted of DMEM (Sigma-Aldrich, catalog no. D5030) supplemented with 20 mM Na-HEPES (pH 7.4) and adjusted to pH 7.4 with NaOH.

### Substrate oxidation assay

Substrate oxidation was measured by CO_2_ trapping, as described in the main text and figure legends. The optimized protocol is outlined here, with additional details in Supporting information (Appendix 1). To measure substrate oxidation in bicarbonate-buffered media, our previously published protocol (14) was modified in several ways (Fig. 3*A*). Following the addition of ^14^C-labeled treatment medium, the gas phase was equilibrated with 5% (v/v) CO_2_ as described above, except a gas trap was installed prior to the initial sealing of the plate. The gas trap was a lid of a 15 ml centrifuge tube (Corning; Sigma-Aldrich, catalog no. CLS430790), drilled to create an 8 mm hole into the center and then turned upside down into the well (14). At the end of the treatment period, 300 μl of 2 M NaOH was dispensed *via* a beveled 27G needle (0.4 × 13 mm; Terumo TMP, catalog no. NN-2713R) into the gas trap, and 100 μl of 3 M perchloric acid was dispensed *via* a beveled 21G needle (0.8 × 38 mm; catalog no. above) to the bottom of the well. The latter quenches the cells and liberates CO_2_ from the medium. The plate was immediately resealed using TopSeal-A PLUS and left at room temperature for at least 1 h. The gas-trapping solution (NaOH) was transferred to 3 ml of Ultima Gold XR scintillant (PerkinElmer, catalog no. 6013119), and radioactivity was measured by liquid scintillation counting with the TriCarb 4910TR (PerkinElmer). Cell-free controls were included to account for any cell-independent signal.

### Isolation of saponifiable lipid

At the end of the treatment period, cells were washed thrice with ice-cold PBS and quenched by freezing. Saponifiable lipids were extracted as described previously (36), except miniaturized for extraction in microfuge tubes. First, cells were scraped in 300 μl of 1 M KOH (prewarmed to 65 °C) and homogenized by pipetting. Each lysate was mixed with 200 μl of ethanol and saponified by incubation at 65 °C for 2 h. Lysates were then acidified by addition of 50 μl of 9 M H_2_SO_4_, and lipids were extracted thrice with petroleum ether (500 μl per extraction). The extracts were combined, washed with 500 μl of water, dried down under N_2_ gas, and resuspended in Ultima Gold XR scintillant. The incorporation of ^14^C-labeled substrate into this extract was measured by liquid scintillation counting and normalized to cell-free controls to account for any cell-independent signal.

### Normalization for cell culture experiments

Unless otherwise specified, data were normalized to protein content, derived from cells cultured in parallel. Cells were washed thrice with PBS, lysed in 1% (v/v) Triton-X100 in PBS, homogenized by pipetting, and clarified by centrifugation for 20 min at 16,000*g* and 4 °C. Protein quantification was performed using the Pierce bicinchoninic acid assay kit (Thermo Fisher Scientific, catalog no. 23227), according to the manufacturer’s instructions.

## Data availability

All data are contained within the manuscript and can be shared upon request, along with an up-to-date version of the optimized protocol: James E. Hudson, +61 7 3362 0141, james.hudson@qimrberghofer.edu.au.

## Supporting information

This article contains supporting information.

## Conflicts of interest

J. E. H. is coinventor on patents for metabolism as a maturation mediator and regenerative target. J. E. H. is a cofounder, scientific advisor, and stockholder in Dynomics.
