# Supplementary material for: A cell culture platform for quantifying metabolic substrate oxidation in bicarbonate-buffered medium
Source: J Biol Chem. 2021 Dec 29;298(2):101547. doi: 10.1016/j.jbc.2021.101547 (PMC8819040; doi:10.1016/j.jbc.2021.101547)
Supplement: Appendix 1 and 2 [file mmc1.docx]

**A cell culture platform for quantifying metabolic substrate oxidation in bicarbonate-buffered medium**

**Supporting Information**

James R. Krycer^1,2^, Mary Lor^1^, Rebecca L. Fitzsimmons^1^, James E. Hudson^1,2,3,^*

^1^ QIMR Berghofer Medical Research Institute

^2^ School of Biomedical Sciences, Faculty of Health, Queensland University of Technology

^3^ School of Biomedical Sciences, Faculty of Medicine, The University of Queensland

* Corresponding author

**Material included in Supporting Information file**

- Appendix 1: Detailed protocol
- Appendix 2: Calculation of CO_2_-trapping capacity of NaOH solution

**References**

1. Krycer, J. R., Diskin, C., Nelson, M. E., Zeng, X. Y., Fazakerley, D. J., and James, D. E. (2018) A gas trapping method for high-throughput metabolic experiments. *Biotechniques* **64**, 27-29

2. Lucile, F., Cézac, P., Contamine, F., Serin, J., Houssin, D., and Arpentinier, P. (2012) Solubility of Carbon Dioxide in Water and Aqueous Solution Containing Sodium Hydroxide at Temperatures from (293.15 to 393.15) K and Pressure up to 5 MPa: Experimental Measurements. *J. Chem. Eng. Data* **57**, 784-789

3. Thompson, J., and Burns, D. A. (2014) Radio-carbon dioxide (^14^CO_2_) trapping and counting. in *Application note: liquid scintillation*, PerkinElmer, Inc., Waltham, MA USA. Document number 007902A_29

4. Krycer, J. R., Elkington, S. D., Diaz-Vegas, A., Cooke, K. C., Burchfield, J. G., Fisher-Wellman, K. H., Cooney, G. J., Fazakerley, D. J., and James, D. E. (2020) Mitochondrial oxidants, but not respiration, are sensitive to glucose in adipocytes. *J Biol Chem* **295**, 99-110

**Appendix 1: Detailed protocol**

In this protocol, our microplate protocol for gas-trapping in microplates (1) has been adapted for bicarbonate-containing medium, as outlined in the accompanying manuscript.

*1. Construction of gas manifold*

Materials required:

- Gas manifold design: https://www.tinkercad.com/things/fGvoG1pJUVb
- PETG (Dremel Digilab, catalog no. PETG-TRA-01)
- 3D45 3D printer (Dremel Digilab)
- Nylon male-male luer-slip connector, x12 (Cole-Parmer, catalog no. 45505-72)
- Bevelled 18G needle, x12 (1.2 x 38 mm; Terumo TMP, catalog no. AN-1838R1)
- M10 barb
- Silicon sealant (RS Components, catalog no. 555-588)
- Araldite (standard heavy duty, RS Components)
- Super glue (e.g., Tarzan’s Grip; Bunnings, catalog no. 1230185)
- Electronic temperature gauge (e.g., Jaycar Electronics, catalog no. QM7209)
- Silicon tubing
- 80% (v/v) ethanol
- Air-flow meter (Dwyer Instruments, catalog no. 172989-00)
- G2 gas cylinder containing 5% (v/v) CO_2_ and 21% (v/v) O_2_ in N_2_ (BOC, catalog no. LSA400011G2)
- 5W heating pad (e.g., Reptile One Heat Mat 240 V, 14 x 15 cm; PetBarn, catalog no. 46527)
- Retort stand and clamp

Procedure:

1. Print the gas manifold using PETG with the 3D-printer, with the following settings: nozzle temperature = 250 °C, build platform temperature = 70 °C, print speed = 60 mm/s.
2. Apply silicon sealant to the upper surface of the base (where it will contact the lid). Adhere the lid and base together, holding them in place with rubber bands. Leave overnight to cure.
3. Adhere the barb to the inlet using araldite glue. Leave overnight to cure.

- *Silicon sealant or super glue can also be used. The barb may need to be trimmed to size.*
- *This can be performed at the same time as the previous step.*

1. Adhere a male-male luer connector to each port using super glue. Leave for at least a few hours to completely cure.

- *Silicon sealant can also be used, but we found that super glue provided stronger adhesion and a better seal.*
- *Make sure the connector is inserted perpendicular to the manifold, otherwise the needles (Step 6) will not be completely parallel to each other.*

1. Adhere a strip of masking tape to an 18G needle using super glue, 1 cm from the tip. Leave for at least a few hours to completely cure.

- *Mark out 1 cm from the tip using a marker pen beforehand.*
- *We found it easiest to apply superglue to the middle of the strip of masking tape, then attach the masking tape to the needle, carefully folding the masking tape without it sliding along the needle.*
- *This can be performed at the same time as the previous step.*
- *This step is optional - we performed it as a precaution to prevent the needle from reaching the bottom of the well as this would lead to contamination of both the needle and cell culture medium.*

1. Fit an 18G needle to each male-male luer connector.
2. Clean the silicon tubing and manifold with 80% (v/v) ethanol by connecting the tubing to the manifold, dispensing 80% (v/v) ethanol into the silicon tubing, and purging with air.

- *Collect the 80% (v/v) ethanol wash in a tray below the manifold.*
- *Let the air run through the system until the system is completely dry.*
- *[Optional] You can check for leaks in the manifold by applying soapy water to the outside of the manifold and checking for bubbles. Leaks may be patched up using silicon sealant.*

1. Using silicon tubing, connect: 1) the inlet of the gas manifold to the outlet of the air-flow meter, and 2) the inlet of the air-flow meter to the gas cylinder.
2. Clamp the manifold to the retort stand.

- *Hold the manifold loosely in the clamp, in preparation for the next step.*

1. Position the heating pad above the manifold, and a layer of aluminium foil above the heat pad. Tighten the clamp.
2. Insert the sensor for the thermometer in between the foil and heating pad, as close to the centre as possible.

*2. Preparation of gas traps*

Materials required:

- 15 ml polypropylene centrifuge tubes (Corning, catalog no. 430790)
- Power drill, with 6 or 8 mm drill bit
- 80% (v/v) ethanol
- Low-lint tissue paper (e.g., Kimwipes)
- Scalpel blade

Procedure:

1. Clean the drill bit with water or 80% (v/v) ethanol.

- *Ensure the drill bit is completely dry before inserting into the drill.*

1. With the lid still attached to the centrifuge tube, drill into the centre of the lid.

- *For safety reasons, ensure the tube is clamped, for instance held in a tube rack.*
- *Make sure that the inner rim of the lid is still intact.*

1. Remove frayed plastic from the hole with a scalpel blade.

- *Place the scalpel blade into the hole, angle the scalpel towards the inner wall of the tube, and rotate the tube.*

1. Clean the top of the lids by wiping with tissue paper soaked with 80% (v/v) ethanol.

*3. Preparation of the cell culture plate*

Materials required:

- 12-well cell culture plate (Corning, catalog no. CLS3513), containing samples of interest and respective cell-free controls. ***Ensure you have parallel samples for cell normalisation (e.g., protein quantification).***
- Treatment medium – see accompanying manuscript for examples of medium to use.
- Gas traps, prepared in Section 2
- Microplate seal (TopSeal-A PLUS, PerkinElemer, catalog no. 6050185)
- Roller or rubber brayer

1. Dispense treatment medium into each well of the culture plate.

- *Dispense the medium close to the bottom of the well so that residual medium does not collect on the side of the well and contaminate the gas trap.*

1. Unscrew a gas trap (perforated tube lid) from each tube and place upside-down into each well of the plate.
2. Repeat steps 1-2 for cell-free controls (i.e., just culture medium), either in adjacent wells on the same plate or another plate in parallel.

- *This is required to account for any cell-independent signal.*

1. Apply plate-seal (to each plate).

- ***Applying a perfect seal is the major pressure point for this protocol.***
- *Trim the plate-seal to size. We found that for the 12-well plates specified in this protocol, trimming 10 mm off the long edge was ideal.*
- *We found leaks occurred substantially less if the tabs were torn off before applying the seal.*
- *Flatten the seal using a soft rubber roller/brayer or any cylindrical object (e.g., tube, cut serological pipette).*
- *[Optional] You can first apply a malleable (yet often breathable) seal such as Breathe-Easy sealing membrane (Sigma-Aldrich, catalog no. Z380059) before applying the TopSeal-A PLUS. This increases the surface area that the TopSeal-A PLUS seal can adhere to.*

*4. Gas equilibration of cell culture plate*

Materials required:

- Gas manifold, prepared in Section 1. ***Ensure the heating pad is turned on and manifold pre-warmed before starting this section.***
- Cell culture plate, prepared in Section 3.
- Microplate seal (TopSeal-A PLUS, PerkinElemer, catalog no. 6050185)
- Roller or rubber brayer
- Bevelled 21G needle (0.8 x 38 mm; Terumo TMP, catalog no. AN-2138R1)
- Laboratory jack (e.g., Eisco 150 x 135 mm; WestLab, catalog no. 071210-0001)

Procedure:

1. Using a 21G needle, make three equidistant holes approximately 0.5 mm from the edge of each well. Flatten the seal again using the roller.
2. Using the laboratory jack, lift the plate up to the gas manifold such that the manifold needles puncture the seal in the middle of each well.

- *Depending on how well the manifold is clamped, you may need one hand on top of the manifold.*
- *Only allow the needle to penetrate the top 1 cm of the well. This can be aided by the masking tape flags prepared in Section 1, Step 5.*

1. Hold the plate in place using the laboratory jack.
2. Equilibrate the gas phase with 5% (v/v) CO2 at 10 L/min for 5 min.

- ***These parameters should be optimised for each individual manifold and if the medium bicarbonate content is varied.*** *This can be achieved by performing the experiments outlined in the accompanying manuscript.*

1. Remove the plate from the manifold and immediately apply another layer of microplate seal.
2. Incubate the plate (without the cell culture lid) as required, for instance 1-2 h at 37 °C.

*5. Quenching and gas-trapping*

Materials required:

- 3 M perchloric acid
- 2 M NaOH
- 1 mL tuberculin syringes (Terumo TMP, catalog no. T3SS01TA)
- Bevelled 27G needles (0.4 x 13 mm; Terumo TMP, catalog no. NN-2713R)
- Bevelled 21G needles (0.8 x 38 mm; Terumo TMP, catalog no. AN-2138R1)
- Microplate seal (TopSeal-A PLUS, PerkinElemer, catalog no. 6050185)
- Roller or rubber brayer
- 6 mL scintillation (pony) vial (PerkinElmer, catalog no. 6000292)
- Ultima Gold XR scintillant (PerkinElmer, catalog no. 6013119)
- TriCarb 4910TR liquid scintillation counter (PerkinElmer)

Procedure:

1. Aspirate NaOH into syringes attached to 27G needles.

- *This and the next step are to ensure the harvesting (subsequent steps) is performed as quickly as possible.*
- *Use 300 µL per well, plus additional ~20% volume spare.*

1. Aspirate perchloric acid into syringes attached to 21G needles.

- *Use 100 µL per well, plus additional ~20% volume spare.*

1. At the end of the treatment period, quickly dispense 300 µL of 2 M NaOH into each gas-trap (prepared in Step 1).

- *A separate syringe/needle may be used for each well – although slower, this minimises cross-contamination.*

1. Quickly dispense 100 µL of 3 M perchloric acid to the bottom of each well using a 21G needle (prepared in Step 2). Gently push the plate back and forth to mix.

- *A cloudy precipitate should appear at the bottom of each well, indicative of acidified protein.*

1. Immediately re-seal the plate with microplate seal.
2. Leave at room temperature for at least an hour, for NaOH to absorb any remaining CO_2_.

- *If the plate is adequately sealed, this can be left overnight.*

1. Remove the microplate seal layers and transfer the NaOH solution to a scintillation vial containing 3 mL of scintillant. Mix by inversion.
2. Measure radioactivity using the scintillation counter.

- *For the TriCarb 4910TR, we use the default ^14^C measurement protocol, but with a 5 min measuring time.*

1. To measure specific activity, dilute an aliquot of treatment medium in scintillant and measure in the scintillation counter.

- *We use a 10 µL aliquot, but this may vary depending on the radioactivity of your medium.*

1. To measure background activity, dilute 300 µL of 2 M NaOH in scintillant and measure in the scintillation counter.
2. To determine the substrate oxidation rate of each sample:
   1. To obtain specific activity, divide the radioactivity content (*A*) of the treatment medium by the amount (*n*) of substrate in the treatment medium (Eq. 1). This should yield DPM per mol of substrate.

$Specific activity (DPM/mol) = A(treatment medium) \div n(substrate in treatment medium)$ (Eq. 1)

- 1. Obtain the radioactivity of the sample, deducting the radioactivity of the cell-free controls (Eq. 2).

$\text{Adjusted sample radioactivity (DPM) = A(sample) }\text{– A(cell-free control)}$ (Eq. 2)

- 1. To obtain the amount of substrate oxidised, divide the sample radioactivity by specific activity (Eq. 3).

$n(substrate oxidised) (mol) = A(Adjusted sample radioactivity) \div specific activity$ (Eq. 3)

- 1. To obtain the substrate oxidation rate, normalise the amount of substrate oxidised to cell number (e.g., protein content used below) and duration of the experiment (Eq. 4).

$Substrate oxidation rate (mol/mg/h) = n(substrate oxidised) \div(protein content \times time)$ (Eq. 4)

*6. Potential safety hazards*

- 3 M Perchloric acid – corrosive
- 2 M NaOH – corrosive
- 80% (v/v) ethanol – flammable
- Ultima Gold XR scintillant – toxic
- 5% (v/v) CO_2_ – asphyxiation hazard
- 18G, 21G, 27G needles – physical hazard
- Scalpel blade – physical hazard
- Power drill – physical hazard
- Adhesives (silicon sealant, araldite, super glue) – toxic
- ^14^C-labelled metabolic tracers – radiation hazard

**Please ensure you complete all necessary risk assessments prior to utilising this protocol.**

**Appendix 2: Calculation of CO_2_-trapping capacity of NaOH solution**

Below is an example calculation for determining whether the NaOH in the gas-trap (300 µL of 2 M NaOH) is adequate for absorbing the CO_2_ released after acidification.

*CO_2_ solubility in NaOH*

In 1 M NaOH, CO_2_ dissolves with a mole fraction of 0.017 at atmospheric pressure (~0.1 MPa) (2). Given the molarity of water is 55 M, thus the solubility of CO_2_ in 1 M NaOH is:

$$0.017 mol/mol \times55 M = 0.935 M$$

Thus, 300 µL of 2 M NaOH will trap:

$$0.935 M \times2 \times300 \mu L = 561 \mu mol of CO\text{2}$$

Another reference provides a more conservative estimate of CO_2_ solubility, stating that 1 M NaOH can trap 0.5 mol CO_2_ per L (3). Using this figure, 300 µL of 2 M NaOH will trap:

$$0.5 mol/L \times2 \times300 \mu L = 300 \mu mol of CO\text{2}$$

*CO_2_ content in the gas phase*

By the ideal gas law:

$$n=\frac{PV}{RT}$$

$$\text{where:}$$

$$n=amount of gas molecules (mol)$$

$$P=101.3 kPa=101.3 \times{10}^{3}\text{ Pa}$$

$$V=V_{well}-V_{medium}=6.9\text{ }\text{cm}^{\text{3}}-0.5 \text{cm}^{\text{3}}=6.4 \text{cm}^{\text{3}}=6.4\times{10}^{-6}\text{ }\text{m}^{\text{3}}$$

$$R=8.314\text{ m}^{\text{3 }}\text{Pa }\text{K}^{\text{-1}} \text{mol}^{\text{-1}}$$

$$T= 310.15 K$$

$$\therefore n=\frac{101.3 \times{10}^{3}\text{ Pa} \times6.4\times{10}^{-6}\text{ }\text{m}^{\text{3}}}{8.314\text{ m}^{\text{3 }}\text{Pa }\text{K}^{\text{-1}} \text{mol}^{\text{-1}}\times310.15 K}\approx2.51\times{10}^{-4}\mathrm{mol}=251 \mu mol$$

Since mol fraction of CO_2_ is 0.05, thus

$$n\left( \mathrm{CO}\text{2} \right)\text{gas}=251 \mu mol\times0.05 mol/mol\approx12.6 \mu mol$$

*CO_2_ content of the medium*

The concentration of NaHCO_3_ in the medium is 44 mM. Thus,

$$n\left( \mathrm{CO}\text{2} \right)\text{medium}=500 \mu L\times44\times{10}^{-3} mol/L= 22 \mu mol$$

*Remaining CO_2_-absorbing capacity*

Using the conservative estimate of CO_2_ solubility, the NaOH can absorb a maximum of 300 µmol based on the above calculations. The capacity of absorbing metabolically-derived CO_2_ is:

$$n\left( \text{CO}\text{2} \right)\text{capacity}=300 \mu mol-12.6 \mu mol-22 \mu mol\approx265 \mu mol$$

*Respiration-derived CO_2_*

We anticipate that respiration of 3T3-L1 adipocytes in a 12-well plate generates CO_2_ at a rate less than 1 µmol/h (4). This concurs with our experiments here (Fig. 3): the maximum glucose oxidation was ~89 nmol/h (100 nM insulin + BAM15, in bicarbonate-buffered medium; glucose oxidation rate presented in Fig. 3 was multiplied by cellular protein content), and glucose oxidation contributes 19.8% of total respiration-derived CO_2_ (4), thus total CO_2_ production is ~450 nmol/h. Either way, for short-term experiments (1-2 h), the CO_2_ derived from cellular respiration is substantially less than the remaining CO_2_-absorbing capacity of the gas-trapping solution.
